# Biological stress, self-rated stress, anxiety and depression in women with takotsubo syndrome

**DOI:** 10.1186/s12872-025-05498-7

**Published:** 2026-01-19

**Authors:** Runa Sundelin, Christina Ekenbäck, Jonas Spaak, Peder Sörensson, Loghman Henareh, Per Tornvall, Patrik Lyngå

**Affiliations:** 1 Department of Clinical Science and Education, Södersjukhuset, Karolinska Institutet, and Cardiology Unit, Södersjukhuset, Stockholm, Sweden; 2 Karolinska Institutet, Department of Clinical Sciences, Division of Cardiovascular Medicine, Danderyd Hospital, Stockholm, Sweden; 3 Department of Medicine Solna, Karolinska Institutet, and Department of Cardiology, Karolinska University Hospital, Stockholm, Sweden; 4 Coronary Artery Disease Area, Heart and Vascular Theme, Karolinska Institute and Karolinska University Hospital, Stockholm, Sweden; 5 Department of Cardiology, Södersjukhuset, Sjukhusbacken 10, Stockholm, S-118 83 Sweden

**Keywords:** Biological stress, Cortisol, Takotsubo syndrome, Stress, Anxiety, Depression

## Abstract

**Aims:**

Takotsubo syndrome is a condition associated with stress, anxiety and depression. Biological stress in takotsubo syndrome is scarcely described in the literature. The aim of this study was to investigate if biological and self-rated stress, anxiety and depression in women with takotsubo syndrome reflect a long-term state of psychological burden, with the hypothesis that saliva cortisol is increased six months after the acute event.

**Method and result:**

Forty-seven women were diagnosed with takotsubo syndrome in a diagnostic cross-sectional study, of those 29 had saliva cortisol measured at awakening and in the evening. Additionally, 34 women completed the perceived stress scale (PSS-14), 35 completed the hospital anxiety and depression scale-anxiety (HADS-A) scale and 33 completed hospital anxiety and depression scale-depression (HADS-D) scale, six months after the acute event. Results were compared with a population-based age-matched group of 227 women from the Swedish cardiopulmonary bioimage study (SCAPIS).

Awakening saliva cortisol levels were significantly increased in takotsubo syndrome patients compared with the SCAPIS group (*p* = 0.039). No significant differences were seen in evening saliva cortisol levels (*p* = 0.267). There was a trend, towards higher self-rated stress (*p* = 0.087), but no significant difference, in anxiety (*p* = 0.133) or depression (*p* = 0.385) between takotsubo syndrome patients and the population-based age-matched group. There were significant correlations between awakening saliva cortisol and anxiety (negative, *p* = 0.036), and between evening cortisol and self-rated stress (positive, *p* = 0.050) but no correlation with depression was observed.

**Conclusion:**

Women with takotsubo syndrome have higher levels of cortisol in saliva measured in the morning, but similar levels measured in the evening six months after the acute event when compared with population-based controls. We found weak associations between biological (cortisol) and psychological (PPS-14, HADS-A and HADS-D) assessments. The effect size of the difference in awakening cortisol was small and the clinical relevance is uncertain, but the results indicates that takotsubo syndrome patients have signs of biological stress. Our findings, together with previous research on stress, anxiety and depression in patients with takotsubo syndrome, indicate a need for further assessment of psychological stress, possibly by interviews, and for secondary prevention to reduce stress.

## Background

Takotsubo syndrome is an acute heart failure syndrome that mimics acute myocardial infarction (AMI) and is associated with reversible left ventricular dysfunction [1, 2]. Nine out of ten patients are women, and most of the women are postmenopausal [3]. A stressful incident of either emotional or physical character is often a trigger for the syndrome [3].

Patients with takotsubo syndrome exhibit higher levels of depressive symptoms and illness-related anxiety, but not general anxiety, compared with healthy controls [4]. This finding aligns with a large cross-sectional study investigating anxiety and depression in patients with AMI at hospital discharge, which reported high levels of self-rated anxiety and moderately elevated depression scores, particularly among women [5]. High levels of stress have been observed when measuring self-rated stress using the perceived stress scale (PSS) in an AMI group, consisting of young women [6]. Recent findings have shown increased levels of anxiety, depression and stress among women with takotsubo syndrome [7], raising the question if takotsubo syndrome is associated with biological stress.

Cortisol is a stress hormone produced in the adrenal glands, which is a part of the hypothalamus–pituitary–adrenal axis (HPAA) [8]. Cortisol levels reach their lowest point around midnight during sleep and then begin to rise and peak 20 to 45 min after awakening. Throughout the day, cortisol levels gradually decline, with minor rises, during exercise or food intake. Following a stressor, it typically takes approximately 20 to 45 min for salivary cortisol levels to reach their peak [9–11]. Elevations in cortisol levels have been associated with several cardiovascular risk factors, including hypertension, hyperlipidemia, and diabetes mellitus [12, 13]. There is a lack of studies addressing awakening salivary cortisol in takotsubo syndrome, with the exception for two small studies [14, 15]. Kastaun et al. [15] showed that 19 women diagnosed with takotsubo syndrome had no significant differences of awakening salivary cortisol levels when compared with age-matched groups of 20 non-ST elevation myocardial infarction (NSTEMI) females, and a group of 20 healthy women. Similar results were demonstrated by Collste et al. [14], when investigating awakening salivary cortisol in 22 patients with takotsubo syndrome compared with a group of 22 healthy individuals. Collste et al. [14] also investigated salivary cortisol levels before going to bed and found no significant differences between the groups.

The primary aim of the present study was to investigate if biological and self-rated stress, anxiety and depression in women with takotsubo syndrome reflect a long-term state of psychological burden, six months after the acute event, with an age-matched group of population-based women. Our hypothesis was that women with takotsubo syndrome have signs of biological stress.

## Methods

Takotsubo syndrome patients were recruited from the Stockholm myocardial Infarction with normal coronaries-2 (SMINC-2) study that was a diagnostic study [16]. Inclusion was made between December 2014 and November 2018 when the following criteria were met: age between 35 and 70, suspected AMI with normal coronaries determined at angiography (coronary angiography without diameter stenosis ≥ 50%), and sinus rhythm at ECG on admission. Exclusion criteria were pulmonary embolism, severe chronic obstructive pulmonary disease, previous myocardial infarction, cardiomyopathy, severe renal impairment, pacemaker, or claustrophobia. All included patients underwent cardiac magnetic resonance (CMR) imaging 2 to 4 days after admission to investigate if a final diagnosis of 70 percent could be achieved [5]. Of the 150 MINOCA patients included into the primary study, 51 were diagnosed with takotsubo syndrome according to CMR, of whom 47 were women [16]. These patients were studied in this secondary analysis. The CMR diagnosis was defined as focal, midventricular, or apical left ventricular wall motional abnormalities with circumferential or segmental myocardial edema, not corresponding to a coronary artery territory, in the absence of corresponding late gadolinium enhancement as described by Eitel and coworkers [17].

The takotsubo syndrome group of women was compared with an age-matched group of 227 generally healthy women from the population-based Swedish cardiopulmonary bioimage study (SCAPIS). The SMINC-2 study is registered at ClinicalTrials.gov. NCT02318498, the 17th of December 2014.

### Cortisol analysis

All patients in SMINC-2 were asked to leave two salivary cortisol samples using Sarstedt salivette kits® six months after the acute event. The first one in the evening, between 6 and 8 pm and the second the following morning, between 6 and 8 am before tooth-brushing. The instruction from Karolinska university laboratory which is, accredited and certified according to ISO 15189, is used to inform patients in the self-collected saliva cortisol procedure when investigating adrenal disorders. The kits were sent home with clear instructions, and the samples were returned during the 6-month follow-up visit at Södersjukhuset in Stockholm. The patients were instructed not to have anything in their mouths, including water or snuff, for 30 min before the test. Compliance with the saliva cortisol sampling instructions was not monitored at the 6-month follow-up visit. The saliva samples were analyzed at the Karolinska university laboratory using the Cisbio-RIA method® until March 6th, 2019, when the analysis method was changed. Consequently, the results from three takotsubo syndrome patients investigated after this date were excluded from the analysis. Another three individuals were excluded from the cortisol analysis because they were taking oral steroids at the time of the saliva cortisol test: two in the takotsubo syndrome group and one in the SCAPIS group. A total of 29 takotsubo syndrome patients donated both evening and awakening salivary cortisol. Identical saliva cortisol instructions were given to the control group from SCAPIS, all samples were collected before the 6th of March 2019, when the analysis method was changed. A total of 205 participants donated awakening cortisol samples, and 206 donated evening cortisol samples.

### Questionnaires

At the 6-month follow-up visit, 34 of the women diagnosed with takotsubo syndrome completed PSS-14, and 35 completed HADS-A, and 33 completed HADS-D. The PSS-14 was completed by 204 participants, the HADS-A by 203 participants, and the HADS-D by 207 participants from the population-based SCAPIS group.

PSS-14 is a self-reported questionnaire measuring levels of stress. Participants estimate how and to what degree they experience their life as unpredictable, uncontrollable, and overloaded in the past month, thus PSS-14 measures the perception of stress. PSS-14 scale consists of 14 items, each with five options ranging from zero to four, with a maximum score of 56. Based on previous studies, a PSS-14 score of ≥ 25 is the cut-off between low and high stress [18, 19]. PSS-14 consists of two subscales: one with negative items reflecting general perceived stress, and one with positive items reflecting coping ability. A recent study has shown that only the subscale measuring perceived stress should be used when assessing general stress. The subscale reflecting coping ability is not recommended for use on its own [20, 21]. The Swedish version of PSS-14 has been shown to be valid in patients with or without known stress-related conditions, with a Cronbachs alpha coefficient of α = 0.84 [22].

The Swedish version of the HAD scale comprises two sub-scales, one assessing anxiety and the other depression. To measure the symptoms of anxiety and depression, we used the scale for anxiety, HADS-A and depression, HADS-D. Each scale consists of seven items related to anxiety or depression, with each item scored from 0 to 3, with four different options. A total score for each scale range from 0 to 21, and the cut off for anxiety or depression is 8 or more [23, 24]. The survey captures the subjects´ feeling over the past week [24]. The questionnaire HADS was found to be accurate in measuring the severity of symptoms in the subscales for both depression and anxiety in the general population [25]. The Swedish version of HADS has shown good validity with a Cronbachs alpha coefficient of 0.84 [23].

### Definition of cardiovascular risk factors

The definition of cardiovascular risk factors was as follows: a current smoker was defined as a person who smoked either occasionally or daily. Hypertension, hyperlipidemia, and diabetes mellitus were defined as having received a diagnosis from a doctor. A psychiatric disorder was defined in the SCAPIS group as currently receiving treatment for any type of psychiatric disorder, whereas in the takotsubo syndrome group, psychiatric disorders were identified based on information from the patients’ medical charts.

### Statistical analysis

Patient and control characteristics were presented as means ± standard deviation (SD) or numbers (percentage). Median and interquartile range (IQR) in box plots were used to display the values of saliva cortisol, HADS-A, HADS-D and PSS-14. The Mann–Whitney U test was used for comparison of continuous data when analyzing salivary cortisol levels and questionnaires between the takotsubo syndrome and SCAPIS groups. Cohen’s d was calculated to support the interpretation of the significant result (*p* = 0.039) for the awakening saliva cortisol, and for the non-significant result (*p* = 0.087) for the PSS-14, in the Mann–Whitney U test. A post-hoc power analysis was performed to assess whether the study had sufficient power for awakening saliva cortisol (*p* = 0.039) and the PSS-14 (*p* = 0.087). Spearman´s rank-order correlation was used to analyze correlations between salivary cortisol levels and questionnaires. Chi-square test was used to compare categorical data. Independent T-test was used when analyzing differences in age. A multinomial logistic regression analysis was performed to investigate the association between the independent variables group (patients/controls), hypertension, hyperlipidemia, and diabetes mellitus, and the dependent variable morning cortisol levels. Morning cortisol levels were divided into tertiles, lowest, middle, and highest. The lowest tertile was used as the reference group in the regression analysis. A significant level of P ≤ 05 was considered. All analysis were performed using statistical package for the social sciences (SPSS) software, version 29.0 and 30.0, except for the post-hoc power analysis, which was conducted using the free statistical software G*Power.

## Results

There were no differences in terms of age between takotsubo syndrome patients and controls. However, there were significant differences in cardiovascular risk factors, such as hypertension, hyperlipidemia, and diabetes mellitus between the takotsubo syndrome group and the control group, but there were no differences in psychiatric disorders between the groups (Table 1).

**Table 1:** General characteristics of patients with takotsubo syndrome compared with population-based controls

	**Takotsubo syndrome**	**Controls**	*P*-values for differences
Participants	47	227	
Women	47	227	
Mean age, SD, years	59 ± 8	57 ± 4	0.118
Present smoker	9 (19)	28 (13)	0.460
Hypertension	17 (37)	46 (20)	**0.016**
Hyperlipidemia	7 (15)	9 (4)	**0.003**
Diabetes mellitus	7 (15)	5 (2)	**< 0.001**
Psychiatric disorders	5 (11)	22 (10)	0.872

Values are presented as means ± standard deviations or numbers (percentages). Significant *P*-values (≤ 0.05) are presented in bold. Psychiatric disorders were obtained from patient records in the takotsubo syndrome group and were self-reported in the SCAPIS group

**Fig. 1:**
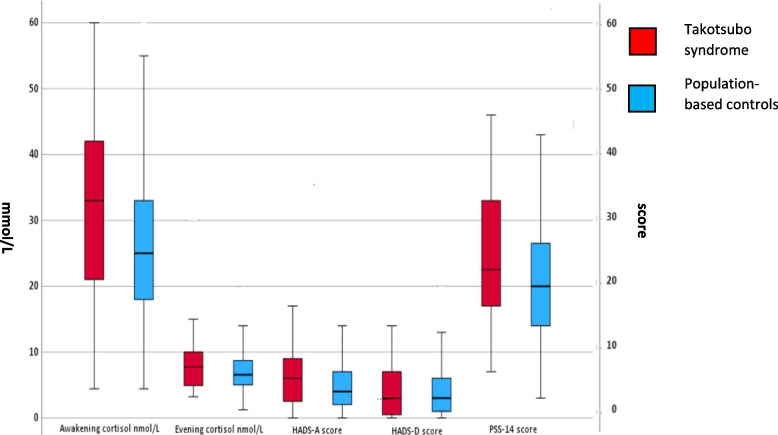
Outliers were excluded only from the figure, not from the analyses. The median is presented for takotsubo syndrome and population-based controls. The boxes indicate the 25th percentile, median, and 75th percentile. The unit for awakening cortisol, and evening cortisol is nmol/L whereas the unit for HADS-A, HADS-D, and PSS-14 is total score

Awakening saliva cortisol levels were approximately 32 percent higher in takotsubo syndrome patients compared with the population-based controls whereas no difference was seen in evening cortisol levels. The result for awakening saliva cortisol levels was significant (*p* = 0.039), and the self-rated stress levels (PSS-14) was non-significant (*p* = 0.087). Both with a small effect size in Cohen’s d, awakening saliva cortisol (Cohen’s d = −0.27), and PSS-14 (Cohen’s d = 0.22), indicating minor differences between the groups, which was also confirmed by the post-hoc analysis, awakening saliva cortisol (45%) and PSS-14 (22%). There were no differences in anxiety (HADS-A) and depression (HADS-D) between takotsubo syndrome patients and controls (Table 2 and Fig. 1). Individuals (patients and controls taken together) with hypertension and diabetes mellitus had higher awakening saliva cortisol levels (*p* = 0.035, respectively *p* = 0.056) than those without these cardiovascular risk factors. There were no differences between individuals with and without hyperlipidemia. Multinomial logistic regression with awakening saliva cortisol tertiles as the dependent variable showed a *p*-value of 0.054 for group (patients/controls) with *p* = 0.851 for hypertension and *p* = 0.626 for diabetes mellitus.

**Table 2:** Cortisol levels, hospital anxiety and depression scale -anxiety (HADS-A), hospital anxiety and depression scale -depression (HADS-D) and perceived stress scale-14 (PSS-14) scores for patients with takotsubo syndrome and population-based controls

	**Takotsubo syndrome**	**Controls**	*P*-values for differences
Awakening cortisol	33.0 (22.5)	25 (15.0)	**0.039**
Evening cortisol	7.7 (5.6)	6.6 (3.7)	0.267
PSS-14	22.5 (17.0)	20.0 (13.0)	0.087
HADS-A	6.0 (7.0)	4.0 (5.0)	0.133
HADS-D	3.0 (4.0)	3.0 (5.0)	0.385

Values are presented as median and (IQR), *N* = 29 for takotsubo syndrome, awakening, and evening cortisol, *N* = 29 for takotsubo syndrome, PSS-14, *N* = 35 for takotsubo syndrome, HADS-A, and *N* = 36 for takotsubo syndrome, HADS-D

Boxplots of summary scores of cortisol levels (left y-axis), hospital anxiety and depression-anxiety (HADS-A), hospital anxiety and depression-depression (HADS-D) and perceived stress scale (PSS-14) (questionnaires right y-axis) for patients with takotsubo syndrome and population-based controls.

A significant negative correlation between awakening cortisol and anxiety (HADS-A) (*p* = 0.036) was seen. Additionally, there was a positive correlation between evening cortisol and stress (PSS-14) (*p* = 0.050). No other correlations were seen (Table 3).

**Table 3:** Correlations between perceived stress scale −14 (PSS-14), respectively hospital anxiety and depression-anxiety (HADS-A) and hospital anxiety and depression-depression (HADS-D) scores with cortisol levels for patients with takotsubo syndrome

	Spearman´s coefficient	*P* value
awakening cortisol/HADS-A	−0.391	**0.036**
awakening cortisol/HADS-D	−0.327	0.160
awakening cortisol/PSS-14	−0.354	0.064
evening cortisol/HADS-A	0.239	0.212
evening cortisol/HADS-D	−0.166	0.485
evening cortisol/PSS-14	0.374	**0.050**

Using Spearman correlation coefficient. *P* ≤.05 is considered as significant

## Discussion

Biological and self-rated stress, anxiety and depression in women with takotsubo syndrome, six months after the acute onset, have been examined together for the first time. The key findings revealed 32 percent higher awakening levels of salivary cortisol compared with an age-matched group of women recruited from the general population. There was a trend towards higher self-rated stress, but no significant difference in anxiety or depression between takotsubo syndrome patients and population-based controls. There were significant differences in cardiovascular risk factors as hypertension, hyperlipidemia, and diabetes mellitus differed between the groups. This could have confounded the results due to glucocorticoid excess associated with cardiovascular risk factors. The finding of a 32 percent higher awakening cortisol level in the takotsubo syndrome group compared to the control group might not be clinically important but is of value for achieving a better understanding of the reason for the syndrome [12, 13]. Furthermore, it is important for the understanding of self-reported stress by PSS-14.

The significant difference with approximately 32 percent higher awakening salivary cortisol levels in takotsubo syndrome patients compared with population-based controls, does not align with findings by Kastaun et al. [15], who conducted a smaller study of 19 takotsubo syndrome females compared with two age-matched control groups. One group consisted of 20 NSTEMI females, and the other group of 20 healthy women. The takotsubo syndrome and NSTEMI group were examined 18 months after the acute onset. Awakening cortisol was measured after awakening, and continuously 15, 30, 45 and 60 min thereafter, with no difference observed between the groups. Collste et al. [13] investigated salivary cortisol levels in 22 takotsubo syndrome patients approximately two years after the acute event and compared the results to an age-matched healthy control group consisting of 22 individuals. Salivary cortisol was measured at awakening in the morning, 30 min after awakening, and before bedtime in the evening. No differences in awakening or evening salivary cortisol levels between the groups were found. The results from both studies differ from the results of our study. Our study consisted of a larger takotsubo syndrome group, and a considerably larger control group recruited from the general population, which likely would lead to a more robust result than the previous two smaller studies. In our study, six months had passed since the acute event, whereas in previous studies [14, 15], the average time intervals were 18 months, respectively two years. It is possible that the passage of time contributed to a reduction in biological stress levels. However, the manual with a time frame given to the patients and the control group for collecting saliva cortisol were not aligned to capture the peak cortisol level, while saliva cortisol peaks approximately 20 to 45 min after awakening, according to the circadian rhythm [9, 11]. The timing to catch the peak of awakening cortisol is of importance. This has been shown when comparing electronically monitored awakening and sampling time to self-reported times. There was a delay in self-reported awakening, which implies that a delay of 5 to 15 min could alter the magnitude and peak timing of awakening cortisol [26]. Thus, the lack of exact timing of saliva sampling in our study could have affected the internal validity of cortisol levels. To improve the accuracy of cortisol measurement, future studies should document the exact time of awakening and ensure that awakening saliva samples are collected within a clearly defined time-window. Moreover, the small effect size highlights the need for larger cohorts to confirm the current findings and to make a firm conclusion regarding the clinical relevance of biological stress in takotsubo syndrome. It is possible that our finding of elevated cortisol upon awakening is false positive, with both the small effect size (Cohen’s d = –0.27) and the post-hoc power estimate of 0.45.

While our results indicated numerically higher self-rated stress levels in takotsubo syndrome patients compared with population-based controls, this difference did not reach statistical significance. The results were examined in a post-hoc analyses, which indicated that the differences seen was small. Our finding replicate the results of a study by Smeijers et al. [27], who reported distress one year after the acute onset using a Likert scale that consisted of elevated tension, stress, anxiety, and feeling of ease (reverse-scored) in 18 takotsubo syndrome patients, 19 patients with heart failure and 19 healthy individuals. High levels of distress were reported among takotsubo syndrome patients without a significant difference compared with healthy controls [27]. Another study demonstrated that self-rated stress levels (PSS-14) were high during the acute onset in a takotsubo syndrome group consisting of 20 patients. The stress levels decreased over time, with no significant change observed between six and twelve months after the acute onset. However self-rated stress levels remained moderate elevated [28]. When studying self-rated stress (PSS-14) in a large group of young women with myocardial infarction at the acute onset and after 12 months [6], the stress levels decreased from high to moderate elevated levels, as also previously shown by our group [28]. Our study was a mixed-method study on stress, using the self-rated PSS-14 questionnaire and qualitative interviews performed six months after the acute event. The interviews confirmed that the patients experienced long-term stress and exhibited higher stress sensitivity than before the acute onset while the questionnaire only showed moderate elevated levels after six months [28]. By combining qualitative and quantitative methods, the findings were strengthened, provided a broader understanding of the patients experience of stress [29]. Theoretically, the self-rated PSS-14 is not sensitive enough and adequate for this group of patients, and takotsubo syndrome patients may underreport their feeling of stress because stress has been present for a long period of their life and maybe stress is the normal state for them. In a large study with over 4,000 participants at baseline and more than 3,000 at two-year follow-up, no correlation was found between perceived stress, measured using the four-item Perceived Stress Scale (PSS-4), and salivary cortisol levels collected at awakening and in the evening. These findings support our results, suggesting that the PSS-14 may not be a reliable instrument for assessing long-term stress, possible due to participants underestimate their stress levels [30]. Qualitative interview studies have shown that patients with takotsubo syndrome often experience long-term stress. Perhaps could qualitative interviews together with questionnaires have reflected the stress and anxiety better, than using questionnaires alone [28]. This psychological distress can be difficult to detect through salivary cortisol measurements as shown previously [14, 15] and in this study. The recurrence rate is approximately 1–2 percent per year [31], however it is unknown if reducing stress, anxiety, and depression in this patient group would result in a reduction in recurrence.

There was a correlation between evening saliva cortisol and self-rated stress (*p* = 0.05), but, surprisingly, no such correlation was found for anxiety or depression. A negative correlation between awakening cortisol and self-rated anxiety (HADS-A) was also observed (*p* = 0.036). This finding is inconsistent with a study involving 47 patients with previous AMI or coronary artery bypass grafting (CABG) and a cut-off score of ≥ 8 on self-rated anxiety (HADS-A) where cortisol levels were measured four times during the first hour after awakening. Significantly higher cortisol levels were observed when self-rated anxiety scores were high compared with when they were moderate, indicate a positive association [32]. However, Vreeburg et al. [33] showed that the correlations between cortisol and anxiety could be conflicting due to differences in subtypes of anxiety, which results in varying cortisol secretion [33]. It is likely difficult to obtain reliable results from our correlations due to the small takotsubo syndrome sample. The lack of strong positive correlations between saliva cortisol and self-rated stress, anxiety or depression also illustrates that the self-rated questionnaires used in the present study measure other dimensions than biological stress. There is also a potential temporal mismatch between saliva cortisol sampling and the questionnaires since cortisol levels reflect the last 24 h diurnal variation, whereas PSS-14 captures perceived stress over the past month and HADS over the past week. There is a need for further research to find questionnaires that better reflect biological stress. Without questionnaires reflecting biological stress, cortisol levels and self-rated stress, anxiety and depression should be interpreted as separate entities in takotsubo syndrome.

### Strengths and limitations

The strengths of this study are the well characterized group of takotsubo patients including a large age-matched control group recruited from the general population, integrating biological (cortisol) and psychological (PSS-14, HADS-A and HADS-D) assessments with analyses at six months, a time frame that is rarely investigated in takotsubo syndrome.

There are major limitations: First, although larger than prior studies on cortisol in takotsubo patients, the sample of 29 women remains relatively small, resulting in modest statistical power (post-hoc estimate 0.45 for awakening cortisol). The findings, particularly the small effect size (Cohen’s d = –0.27), should therefore be interpreted with caution.

Second, the limited numbers of cortisol samples per subject and the written instructions given to the takotsubo syndrome patients and controls regarding the collection of the salivary cortisol samples. It was not specified that the samples should align with the circadian rhythm to catch the cortisol peak, instead a time frame was given. Without electronically verified sampling times, there is risk of misclassification and underestimation of true awakening cortisol response. This methodological issue reduces internal validity. Third, the control group differed significantly from the takotsubo group in cardiovascular risk factors (hypertension, hyperlipidemia, diabetes mellitus), which are known to influence cortisol regulation. Although regression analyses were performed, residual confounding remains likely. Fourth, psychiatric diagnoses were derived from medical records in patients but self-reported in controls, potentially introducing bias or underreporting. Furthermore, the study relies exclusively on self-reported questionnaires (PSS-14, HADS-A, HADS-D). These tools may not fully capture long-term stress burden in takotsubo patients, who may normalize chronic stress. The absence of qualitative interviews or more sensitive psychometric instruments limits interpretability. Fifth, the study group was restricted to women aged 35–70 with non-obstructive coronary arteries, excluding men, elderly patients, or those with major comorbidities. Generalizability is therefore limited. Finally, the takotsubo diagnosis was based on CMR criteria rather than standardized Mayo or InterTAK criteria. While justified, this may slightly limit comparability with other studies.

## Conclusion

Women with takotsubo syndrome have higher levels of cortisol in saliva measured in the morning, but similar levels measured in the evening six months after the acute event when compared with population-based controls. We found weak associations between biological (cortisol) and psychological (PPS-14, HADS-A and HADS-D) assessments. The effect size of the difference in awakening cortisol was small and the clinical relevance is uncertain, but the results indicates that takotsubo syndrome patients have signs of biological stress. Our findings, together with previous research on stress, anxiety and depression in patients with takotsubo syndrome, indicate a need for further assessment of psychological stress, possibly by interviews, and for secondary prevention to reduce stress.

## Data Availability

The datasets were used for analysis in accordance with the statistical methods applied in the current study, and the de-identified data are available from the corresponding author upon reasonable request, in alignment with the FAIR data principles.
